# Short hairpin RNA targeting Twist1 suppresses cell proliferation and improves chemosensitivity to cisplatin in HeLa human cervical cancer cells

**DOI:** 10.3892/or.2012.1633

**Published:** 2012-01-12

**Authors:** KEXIU ZHU, LIHONG CHEN, XIAOBING HAN, JIA WANG, JUE WANG

**Affiliations:** 1 Department of Obstetrics and Gynecology, First Hospital, Xi’an Jiaotong University, Xi’an, Shaanxi 710061; 2 Department of Obstetrics and Gynecology, Shaanxi Provincial People’s Hospital, Xi’an, Shaanxi 710068; 3 The Key Laboratory of Biomedical Information Engineering of the Ministry of Education, Research Center of Rehabilitation Science and Technology, School of Life Science and Technology, Xi’an Jiaotong University; 4 National Engineering Research Center of Health Care and Medical Devices, Xi’an Jiaotong University Branch, Xi’an, Shaanxi 710049, P.R. China

**Keywords:** cervical cancer, drug-resistance, Twist1, MDR1/P-gp, cisplatin

## Abstract

Development of multidrug resistance (MDR) remains a major hurdle to successful cancer chemotherapy and MDR1/P-gp overexpression is believed to be mainly responsible for MDR of tumor cells. Twist1, which is a highly conserved transcription factor that belongs to the family of basic helix-loop-helix proteins, has been shown to be a major regulator of the epithelial-mesenchymal transition (EMT), and therefore promotes carcinoma metastasis. Recently, a novel function of Twist1 was reported to confer radioresistance or chemoresistance in cervical cancer. However, mechanisms of such efficacy are not completely elucidated. In the present study, we firstly analyzed the relationship between Twist1 and MDR1/P-gp expression in human cervical cancer specimens and demonstrated a positive correlation between Twist1 and MDR1/P-gp expression in the same patient. Additionally, we provide the first evidence that silencing of Twist1 by RNAi downregulated MDR1/P-gp expression in HeLa cervical cancer cells, suppressed the cell proliferation, inhibited Rhodamine123 efflux activity of cells and sensitized cells to cisplatin treatment. Collectively, these findings suggest that Twist1-mediated modulation of MDR1/P-gp expression plays an important role in sensitization of cervical cancer cells to cisplatin, and also indicate a novel therapeutic strategy to overcome drug resistance through inactivation of Twist1 expression in cervical cancer.

## Introduction

Cervical cancer is the third most commonly diagnosed cancer and the fourth leading cause of cancer-related death in females worldwide, accounting for 9% (529,800) of the total new cancer cases and 8% (275,100) of the total cancer deaths among females in 2008 (1). Surgery, alone or combined with radiotherapy has long been the cornerstone in the treatment of cervical cancer (2,3). Unfortunately, not all patients respond to such treatment, leading to an extremely low survival rate for advanced stage. To increase survival rates, different strategies with neoadjuvant chemotherapy have been developed (4,5). Additionally, cisplatin-based chemotherapy has traditionally been reserved as part of treatment for metastatic or recurrent cervical cancer disease (6). However, resistance of cervical cancer cells to antineoplastic agents is the major reason why chemotherapy-based treatment modalities of malignant tumors may fail (7,8). Therefore, exploring a novel approach to improve drug sensitivity appeared to be urgently needed.

Accumulating evidence has demonstrated that cervical cancer cells can exhibit a cross-resistant phenotype against several unrelated drugs that differ widely with respect to molecular structure and target specificity (9,10). This phenomenon has been termed multidrug resistance (MDR). MDR has most often been linked to overexpression of MDR1/P-gp, which is overexpressed in many drug-resistant cell lines and in cervical cancer (11). MDR1/P-gp functions as a xenobiotics pump transporting a variety of toxic agents including anticancer drugs from the intracellular milieu to the outside of the cell (12,13). In view of the vital role of MDR-1/P-gp in the treatment of cancer, prevention of MDR-1/P-gp induction in cancer cells may help to avert drug resistance.

Twist1, a basic helix-loop-helix transcription factor, has been known to contribute to tumor metastasis by promoting an epithelial-mesenchymal transition (EMT), which is a process initially observed in embryonic development in which cells lose epithelial characteristics and gain mesenchymal properties to increase motility and invasion (14,15). Recently, a bulk of evidence has demonstrated a close link between EMT and insensitivity to several growth factors or chemotherapeutic agents (16,17). A novel function of Twist1 has been reported in the development of acquired chemoresistance in human cancer cells. Upregulation of Twist1 was associated with cellular resistance to microtubule-targeting anticancer drugs in various types of cancers (18–20). In addition, overexpression of Twist1 confers radioresistance or chemoresistance of cervical cancers, thereby leading to a poorer prognosis (21). Therefore, targeting Twist1 could be a novel therapeutic approach for the treatment of cervical cancer by overcoming drug resistance.

These above observations prompted us to examine whether Twist1 acts as a potential regulator of MDR1/P-gp. Therefore, the purpose of this study was to investigate the relationship of Twist1 and MDR1/P-gp in cervical cancer and to explore whether Twist1 played an important role in drug resistance of cervical cancer cells by regulating MDR1/P-gp. To the best of our knowledge, this is the first report to describe that the expression of MDR1/P-gp was significantly positively associated with Twist1 expression in clinical cervical cancer tissues and that Twist1 silencing not only downregulated MDR1/P-gp expression but also enhanced chemosensitivity of human cervical cancer HeLa cells to cisplatin.

## Materials and methods

### Cell line and tissue specimens

The human cervical cancer HeLa cell line (obtained from ATCC) was cultured in Dulbecco’s modified Eagle’s medium (DMEM) supplemented with 10% fetal bovine serum (FBS) at 37°C in a 5% CO_2_ atmosphere. Forty-two human cervical cancer (including 26 squamous cell carcinoma and 16 adenocarcinoma) tissue specimens were obtained from patients who underwent surgical resection with the approval of the Institutional Review Board (IRB) of the First Affiliated Hospital of Medical College of Xi’an Jiaotong University.

### Immunohistochemistry (IHC)

IHC was conducted using a Dako Autostainer Plus system (Dako Corp., Carpinteria, CA) according to the manufacturer’s protocol. Formalin-fixed, paraffin-embedded cervical tissues were cut at a thickness of 4 μm, then were deparaffinized, rehydrated and subjected to 5-min pressure-cooking antigen retrieval in citrate buffer (10 mM, pH 6.0), 10 min double endogenous enzyme block, 60 min mouse anti-Twist1 (Abcam, 1:50) or mouse anti-MDR1/P-gp (Santa Cruz Biotechnology, 1:100) primary antibody incubation and 30-min incubation with DakoCytomation EnVision HRP reagent for mouse antibodies. Signals were detected by adding the substrate hydrogen peroxide using diaminobenzidine (DAB) as a chromogen followed by a 45-sec hematoxylin counterstaining. As a negative control, the primary antibody was replaced with normal IgG at an appropriate dilution. All of the immunostained slides were analyzed by two histopathologists. A case was diagnosed as positive when Twist1 staining in the nuclei and cytoplasm and the MDR1/P-gp staining in the cytoplasm and membrane were observed in >10% cells.

### Cell transfection and clone selection

Chemically-synthesized pGPU6/GFP/Neo vectors containing short hairpin RNA (shRNA) against *Twist1* (GenBank accession no. NM_000474) mRNA pGPU6/GFP/Neo-Twist1 (sh-Twist1) (F: 5′-CACCG GTACATCGACTTCCTCTACCTTCAAGAGAGGTAGAGG AAGTCGATGTACCTTTTTTG-3′; R: 5′-GATCCAAAAAA GGTACATCGACTTCCTCTACCTCTCTTGAAGGTAGAG GAAGTCGATGTACC-3′; target sequence: 5′-GGTACATC GACTTCCTCTACC-3′) and pGPU6/GFP/Neo vectors containing negative control shRNA (sh-NC) were purchased from Shanghai Genepharma Co. (Shanghai, China). For transient transfection, HeLa cells were seeded in 6-well plates at the density of 5×10^4^ cells/well and incubated at 37°C in an atmosphere with 5% CO_2_ for 12 h. For each well, 7 μl of Fugene HD Transfection Reagent (Roche) and 2 μg of sh-Twist1 or sh-NC mixed together were diluted into 100 μl of DMEM culture medium without serum and incubated for 20 min. Subsequently, the mixture was added to the cells. Six hours later, the medium was replaced with 2 ml of fresh DMEM medium containing 10% FBS, and subsequent experiments were performed after culturing for another 24 h. For stable transfection, cells were cultured in DMEM culture medium for 48 h after transfection as above, and then cells were grown in 10-cm cell plates with medium containing 300 μg/ml G418 (Invitrogen). After 3 weeks of culture, visible colonies were picked up and expanded. The stably transfected clones of HeLa cells (HeLa/sh-Twist1 and HeLa/sh-NC) were observed to show green fluorescence under microscope. Western blotting was used to identify the positive clone.

### Cell viability assay

Cell viability and IC_50_ values (drug concentration causing 50% inhibition of cell growth) were analyzed by the methyl tetrazolium (MTT) assay. For transient transfection, cells were seeded at 5×10^3^ cells/well in 96-well plates one day before transfection, then the MTT assay was performed just before transfection as well as at 24, 48 and 72 h after transfection. For stably transfected clones, cisplatin at various concentrations (from 0 to 50 μM) was added to each 96-well plate and the MTT assay was performed at 48 and 72 h after intervention. For the assay, 20 μl of 5 mg/ml MTT (Invitrogen) was added to each well, then the cells were incubated for 4 h before 180 μl dimethylsulphoxide (DMSO, Sigma) was added. After the insoluble crystals were completely dissolved, the absorbance of each well was measured at 490 nm using a microplate reader (Bio-Rad Laboratories, Hercules, CA, USA).

### Apoptosis assay

HeLa cells were harvested 48 h after sh-Twist1 or sh-NC transfection and resuspended in binding buffer (100 μl/500,000 cells: 140 mmol/l NaCl, 5 mmol/l CaCl_2_, and 10 mmol/l HEPES buffer) followed by 3 washes with phosphate-buffered saline (PBS, pH 7.4). Annexin-V-FITC (5 μl) and 10 μl propidium iodide (PI, 1 μg/ml) were added and then the cell suspension was incubated in a dark chamber at room temperature for 10 min. After centrifugation, the cell pellet was resuspended in 200 μl binding buffer and subjected to FACS analysis using the FACSort flow cytometer (BD Biosciences). The percentage of apoptotic and necrotic cells was determined using FCS express software (DeNovo Software, Los Angeles, CA).

### Reverse transcription-polymerase chain reaction (RT-PCR)

After shRNA transfection, total RNA was isolated using the RNAfast200 Total RNA Extract kit (Fastagene, China). The RNA (2 μg) was reverse transcribed using the RevertAid™ First Strand cDNA Synthesis kit (MBI Fermentas, Germany) according to the manufacturer’s instructions. All PCR analyses were subsequently performed with 1 μg of the cDNA reaction utilizing conditions as follows: 94°C, 5 min; 32 cycles of 94°C, 30 sec; 58°C, 30 sec; 72°C, 45 sec. Reactions were terminated with 72°C, 7-min extension. Primers used were F: 5′-CGTTTC CAGGAGGCCTGGCG-3′ and R: 5′-GCGAGACTGGCGA GCTGGAC-3′ for Twist1 and for β-actin F: 5′-GGCGGCACC ACCATGTACCCT-3′ and R: 5′-AGGGGCCGGACTCGT CATACT-3′). PCR products were analyzed by 2% agarose gel electrophoresis and visualized using ethidium bromide staining.

### Western blotting

After the transfection, cells were lysed in ice-cold RIPA lysis buffer (1% NP-40, 0.1% SDS, 0.5% sodium deoxycholate, 150 mmol/l NaCl and 10 mmol/l Tris-HCl) containing a protease inhibitor cocktail. A total of 30 μg of protein was separated by 10% SDS-PAGE and transferred to nitrocellulose membranes. After being blocked in Tris-buffered saline (TBS) containing 5% skim milk at room temperature for 1 h, the membranes were incubated with mouse monoclonal Twist1 antibody (Abcam, 1:200) or mouse monoclonal MDR1 antibody (Santa Cruz Biotechnology, 1:250) at 4°C for 12 h, and then with horseradish peroxidase (HRP)-conjugated anti-mouse antibody (Zhongshan, China) at a dilution of 1:3,000 at room temperature for 1 h. Signals were detected on X-ray film using the ECL detection system (Pierce, Rockford, IL, USA). Loading differences were normalized using a monoclonal GAPDH antibody.

### Immunofluorescence staining

HeLa/sh-NC and HeLa/sh-Twist1 cells were cultured on glass coverslips for 24 h. The cells were washed with PBS and fixed with 4% paraformaldehyde for 20 min, permeabilized with 0.1% Triton X-100 and blocked with 3% bovin serum albumin (BSA) for 1 h. Coverslips were incubated with Twist1 or MDR1/P-gp primary antibodies described above (dilution rate for both antibodies used were 1:100) at 4°C overnight and then with TRITC or FITC labeled secondary IgG conjugates (Zhongshan, China). Atfer 4,6-diamidino-2-phenylindole (DAPI, 1 μg/ml, Sigma) counterstaining for nuclei, fluorescence was visualized by fluorescence microscopy (Olympus Optical Co., Ltd., Tokyo, Japan).

### Detection of MDR1/P-gp function

Efflux of rhodamine 123 (Rh123) was chosen to detect the MDR1/P-gp function of cells. HeLa/sh-NC and HeLa/sh-Twist1 cells were cultured in 6-well plates, and when the cells reached 70–80% confluence, Rh123 (Sigma) was added to the cells at a final concentration of 0.25 μg/ml and incubated at 37°C for 1 h. Cells were washed 3 times with 4°C PBS and resuspended at 5–10×10^5^ cells/ml in 4°C PBS. Rh123 fluorescence was analyzed with a FACStar flow cytometer (BD Biosciences) equipped with an argon laser. The blast population was gated by forward and side scatter characteristics. Rh123 fluorescence of 10,000 cells was measured logarithmically through a 530 nm bandpass filter at an excitation wavelength of 488 nm. HeLa cells without incubation with Rh123 served as a negative control. Rh123 efflux was measured by counting cells in the M1 region of the plot and calculated as the percentage of cells in the M1 region of the plot. The higher the percentage of cells in the M1 region the greater the cellular Rh123 efflux and also the greater the MDR1/P-gp function.

### Statistical analysis

All statistical analyses were performed using SPSS 16 (SPSS Inc., Chicago, IL). Statistical significance of differences among the control and various treatment groups were compared by one-way ANOVA, followed by the Dunnett’s t-test for separate comparisons. The expression of Twist1 and MDR1/P-gp were compared using the Pearson’s test. P<0.05 was considered statistically significant.

## Results

### Expression of Twist1 and MDR1/P-gp in clinical cervical carcinoma tissues

**Figure 1:**
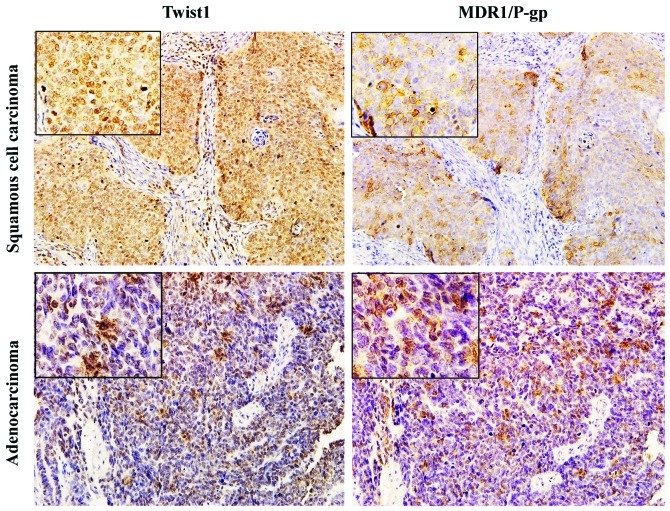
Immunohistochemical staining for Twist1 and MDR1/P-gp proteins in human cervical cancer samples. Samples were subjected to immunohistochemical staining with antibodies against Twist1 and MDR1/P-gp, respectively. Representative results of the Twist1 and MDR1/P-gp staining in serial sections from squamous cell carcinoma samples (upper panel) and adenocarcinoma samples (lower panel) showing nuclear and/or cytoplasmic staining for Twist1, membrane and/or cytoplasmic staining for MDR1/P-gp in the same field. Images were captured at magnifications of ×200 and the inset shows higher magnification of ×400.

Immunohistochemical staining showed strong cytoplasmic and/or nuclear staining for Twist1, cytoplasmic and/or membrane staining for MDR1/P-gp in both cervical squamous cell carcinoma and adenocarcinoma tissues (Fig. 1). According to the total 42 tissue specimens, the positive expression of Twist1 and MDR1/P-gp was 31/42 (73.8%) and 27/42 (64.3%), respectively. Of the 31 Twist1-positive specimens, 24 (77.4%) showed positive staining of MDR1/P-gp. Additionally, of the 11 Twist1-negative specimens, 8 (72.7%) were MDR1/P-gp negative. The Spearman analysis showed that the expression level of MDR1/P-gp was significantly positively associated with Twist1 expression (R=0.460, P=0.008; Table I).

### shRNA silencing of Twist1 at the mRNA and protein levels

**Figure 2:**
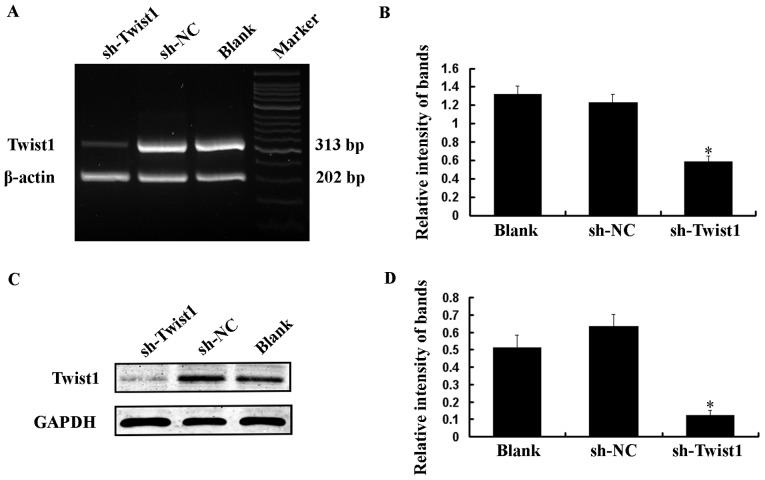
Ablation of endogenous Twist1 expression in HeLa cells using shRNA. HeLa cells were not transfected (Blank), transfected with control shRNA (sh-NC) or shRNA targeting Twist1 (sh-Twist1) for 48 h, and then RT-PCR (A) and western blotting (C) were performed to detect Twist1 mRNA and protein levels. β-actin and GAPDH served as loading controls. sh-Twist1 significantly ablated endogenous Twist1 at both the mRNA and protein levels. Quantitative analyses are shown (B and D) in the right panels and the data represent the mean ± SEM of three independent experiments. ^*^P<0.05 vs. the Blank.

HeLa cells, which have a high level of Twist1 expression, were transiently transfected with 1 μg/ml sh-Twist1 or sh-NC. Total RNA and protein were isolated and analyzed by RT-PCR and western blotting at 48 h after transfection. Compared with the Blank (no shRNA) and sh-NC transfected cells, the expression of Twist1 was obviously suppressed in cells transfected with sh-Twist1 at both the mRNA and protein levels (Fig. 2).

### Downregulation of Twist1 inhibits growth and promotes apoptosis of HeLa cells

**Figure 3:**
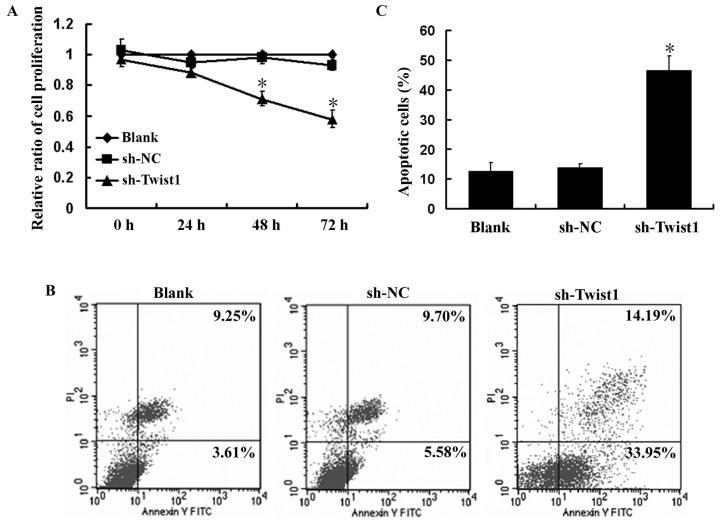
Growth inhibition and apoptosis induction of sh-Twist1 in HeLa cells. (A) The MTT assay was performed to assess the growth of HeLa cells untransfected, transfected with sh-NC or sh-Twist1 for 0, 24, 48 and 72 h. The relative ratio of cell proliferation to untransfected cells was measured and the data shown are the mean ± SEM of three independent experiments. ^*^P<0.05 vs. the Blank. (B) Representative FACS analyses for apoptosis induction of sh-Twist1 on HeLa cells. Untransfected, sh-NC or sh-Twist1 transfected cells were harvested at 48 h and stained with Annexin-V-FITC and PI followed by FACScan flow cytometric analysis. The lower right (LR) quadrant of the FACS histogram indicates the percentage of early apoptotic cells (Annexin-V-FITC stained cells) and the upper right (UR) quadrant indicates the percentage of late apoptotic cells (Annexin-V-FITC and PI-stained cells). (C) Quantitative analysis of the population of total apoptotic cells. Three independent experiments were conducted and the data shown are the mean ± SEM. ^*^P<0.05 vs. the Blank.

The role of Twist1 in the growth of HeLa cells was determined by MTT assay. As shown in Fig. 3A, downregulation of Twist1 by transient transfection of sh-Twist1 in HeLa cells caused significant inhibition of cell proliferation compared with the Blank and sh-NC transfected cells (P<0.05). The proliferation rate of HeLa cells transfected with 1 μg/ml sh-Twist1 was 0.88±0.032, 0.71±0.046 and 0.58±0.039 for 24, 48 and 72 h, respectively, which indicated a time-dependent growth inhibition effect.

Since we observed the inhibitory effects of Twist1 silencing on the growth of HeLa cells, we next examined the effects of Twist1 silencing on the apoptosis of HeLa cells using Annexin-V and PI double staining. The effects of Twist1 silencing on the apoptosis of HeLa cells, as measured by flow cytometry, are shown in Fig. 3B and C. sh-Twist1 resulted in 48.14% of apoptotic cells, while the baseline of the control cells (Blank) was 12.86% (P<0.05). These results indicate that downregulation of Twist1 inhibited growth and promoted apoptosis in HeLa cells.

### Silencing of Twist1 represses MDR1/P-gp expression

**Figure 4:**
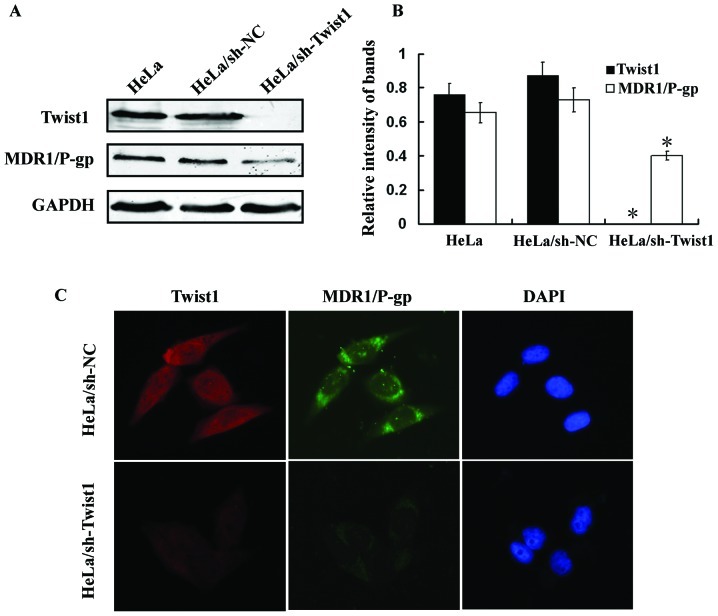
Downregulation of MDR1/P-gp by Twist1 silencing in HeLa cells. Two stably transfected clones of HeLa cells, HeLa/sh-NC and HeLa/sh-Twist1, were established as described in Materials and methods. (A) Cell lysates were subjected to SDS-PAGE followed by western blotting using specific antibodies against Twist1 and MDR1/P-gp. GAPDH served as a loading control. HeLa/sh-Twist1 cells showed no expression of Twist1 and decreased expression of MDR1/P-gp. (B) Quantitative analyses for protein levels were shown and the data represent the mean ± SEM of three independent experiments. ^*^P<0.05 vs. parental HeLa cells. (C) Immunofluorescence staining of Twist1 and MDR1/P-gp in HeLa/sh-NC and HeLa/sh-Twist1 cells. Cells were double-stained for Twist1 (red) and MDR1/P-gp (green). Nuclei were counterstained blue with DAPI. HeLa/sh-NC cells showed strong cytoplasmic and nuclear staining for Twist1, cytoplasmic and membrane staining for MDR1/P-gp, whereas HeLa/sh-Twist1 cells showed weak Twist1 and MDR1/P-gp staining.

To further investigate the relationship between Twist1 and MDR1/P-gp in cervical cancer, stable Twist1 knockdown (HeLa/sh-Twist1) and control (HeLa/sh-NC) HeLa cells were generated. The protein expression of Twist1 and MDR1/P-gp were then investigated by western blotting. As shown in Fig. 4A and B, Twist1 ablation in HeLa/sh-Twist1 cells accompanied with a significant repression on MDR1/P-gp expression was observed. Immunofluorescence staining confirmed these findings (Fig. 4C).

### Silencing Twist1 abates MDR1/P-gp function

**Figure 5:**
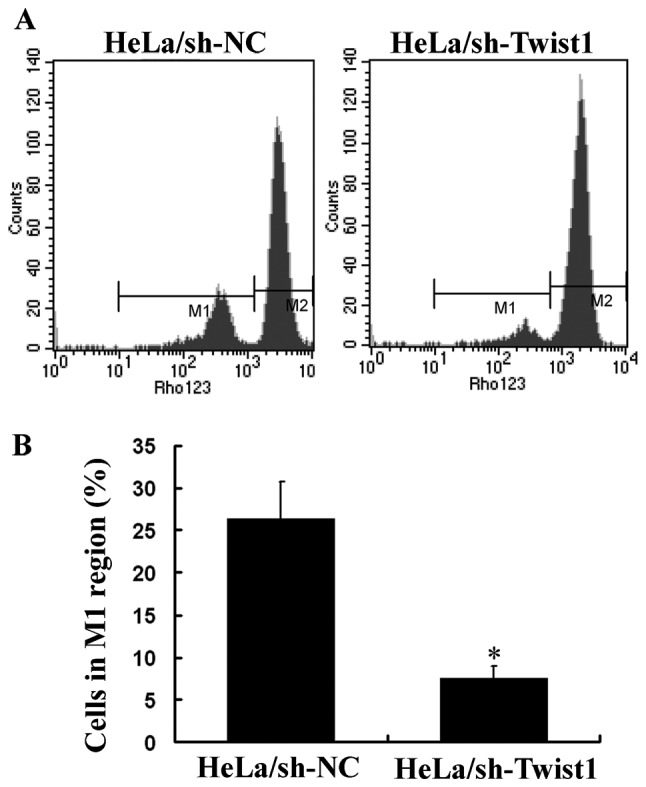
Effect of Twist1 silencing on MDR1/P-gp activity in HeLa cells. (A) Efflux of Rh123 was chosen to detect the MDR1/P-gp function of HeLa/sh-NC cells and HeLa/sh-Twist1 cells. Cells were incubated with 0.25 μg/ml Rh123 for 1 h at 37°C and then Rh123 fluorescence was analyzed by FACStar flow cytometry. Rh123 efflux was measured by counting cells in the M1 region of the plot. The marker bar M1 was set to indicate the cells with high Rh123 efflux and M2 was set to indicate the cells with low Rh123 efflux. (B) Quantitative analysis of the percentage of M1 cells. Three independent experiments were performed and data shown are the mean ±SEM. ^*^P<0.05 vs. the HeLa/sh-NC cells.

Since MDR1/P-gp was downregulated by Twist1 ablation, we next examined the effects of silencing Twist1 on MDR1/P-gp function. Rh123 efflux is sensitive and specific for indicating the transport function of MDR1/P-gp. MDR1/P-gp-mediated transport indicated by intracellular decrease of Rh123 fluorescence was studied using flow cytometry. Compared with HeLa/sh-NC cells, a significant increase of intracellular Rh123 was observed in HeLa/sh-Twist1 cells, the mean percentage of Rh123 efflux cells (cells in M1 region) was 26.41% and 7.53% in HeLa/sh-NC cells and HeLa/sh-Twist1 cells, respectively (P<0.05) (Fig. 5). These results suggest that the repression of MDR1/P-gp expression by silencing Twist1 in HeLa/sh-Twist1 cells results in a great lost of MDR1/P-gp function in drug efflux.

### Abated MDR1/P-gp function increases cell sensitivity to cisplatin

**Figure 6:**
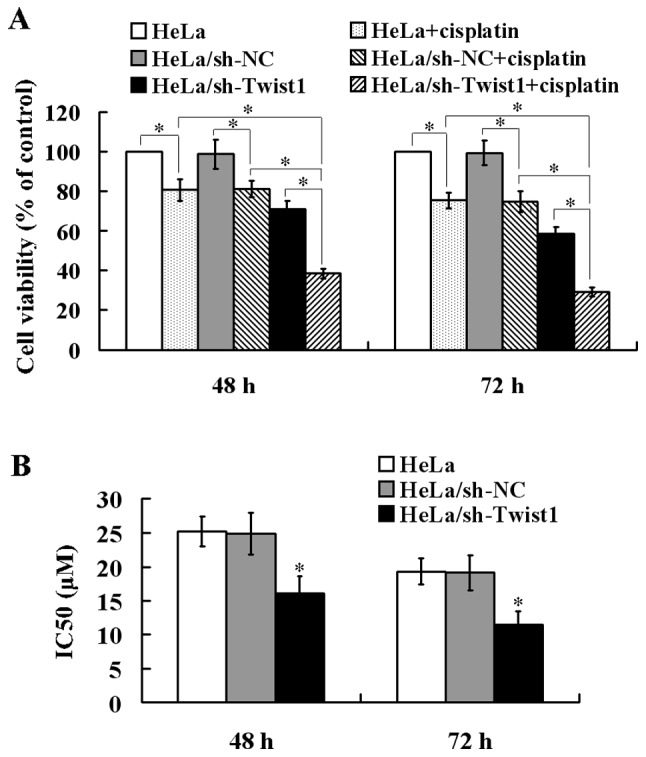
Increased sensitivity to cisplatin in Twist1 silencing HeLa cells. (A) Effect of cisplatin on cell viability. HeLa cells, HeLa/sh-NC cells and HeLa/sh-Twist1 cells were treated with 15 μM cisplatin for 48 and 72 h, respectively, cell viabilities were then determined by the MTT assay. HeLa/sh-Twist1 cells showed a decreased viability in response to cisplatin. (B) IC_50_ values of cisplatin. Cells were treated with various concentrations of cisplatin for 48 and 72 h, and cell growth were also determined by the MTT assay. The IC_50_ values were estimated from growth inhibition curves, showing a significant decrease of IC_50_ for cisplatin in HeLa/sh-Twist1 cells. All of the data shown represent the mean ± SEM of three independent experiments. ^*^P<0.05.

To evaluate the biological significance of Twist1 on the cell sensitivity to cisplatin, MTT assay was then performed. Compared with HeLa and HeLa/sh-NC cells, HeLa/sh-Twist1 cells showed a significant decrease in cell viability over 48 and 72 h. Cisplatin (15 μM) induced only a moderate (but significant) decrease in cell viability of HeLa cells and HeLa/sh-NC cells, but this decrease was much more significant in HeLa/sh-Twist1 cells (Fig. 6A). The IC_50_ value to cisplatin for HeLa cells, HeLa/sh-NC cells and HeLa/sh-Twist1 cells was 25.2±2.79, 24.9±1.84, 16.1±1.98 μM and 19.3±2.31, 18.7±1.92, 11.4±2.01 μM for 48 and 72 h, respectively, suggesting the reduced IC_50_ value to cisplatin by Twist1 silencing (Fig. 6B).

## Discussion

Recently, a novel function of Twist1 has been reported in the development of acquired chemoresistance in human cancer cells (18–20). However, the reason that Twist1 contributes to drug resistance in the treatment of cervical cancer has not yet been established. In this study, we firstly analyzed the relationship between Twist1 and MDR1/P-gp expression in cervical cancer specimens and demonstrated a positive correlation between Twist1 and MDR1/P-gp expression in the same patient. Additionally, we provided the first evidence that silencing of Twist1 downregulated MDR1/P-gp expression, inhibited its efflux activity, and sensitized cervical cancer cells to cisplatin treatment.

Twist1 is a highly conserved transcription factor that belongs to the family of basic helix-loop-helix proteins, which play a central role in cell type determination and cell differentiation (14,15). Recently, accumulating studies have shown that Twist1 was overexpressed in a variety of solid cancers including breast, prostate and gastric carcinomas rendering Twist1 as a potential oncogene in tumorigenesis (22–24). Twist1 has been found to function as an antiapoptotic factor through both p53-dependent and p53-independent pathways (22). Downregulation of Twist1 through small interfering RNA promotes apoptosis in human breast cancer and melanoma cell lines (25). Consistent with these findings, we also observed that inactivation of Twist1 by RNA interference induced cell apoptosis in cervical cancer HeLa cells though the mechanisms have not been studied.

EMT is a complex process which disaggregates structured epithelial units to enable cell motility and morphogenesis in embryonic development and is critically linked with up-regulated invasion, metastasis and angiogenesis in cancer progression (14,15). In addition, the EMT of cancer cells not only causes increased metastasis, but also contributes to drug resistance (19). Furthermore, it has been reported that drug-resistant MCF-7 cells exhibit EMT gene expression patterns and paclitaxel-resistant epithelial ovarian carcinoma cells lose their epithelial features, acquire mesenchymal characteristics, and increase their capacity for migration and invasion, which are events characteristic of the EMT (16). Twist1 is a major regulator of EMT and it has also been identified as capable of promoting carcinoma metastasis. The elevated Twist1 expression is found to be positively correlated with aggressiveness of cancer and poor survival rate (22,24). Recently, Li *et al* demonstrated that cells undergoing EMT displayed up-regulation of MDR1/P-gp, MDR to chemotherapeutic agents as well as increased *in vitro* invasiveness potential (19). Twist1 RNAi largely inhibited EMT induction and partially reversed MDR phenotype (19). However, the relationship between EMT and drug resistance seems complicated. Whether the expression of MDR1/P-gp is regulated by Twist1 remains to be elucidated.

The expression of MDR1/P-gp is regulated at the transcriptional level by multiple signaling pathways (26), including those mediated by hypoxia-inducible factor-1α (HIF-1α), p53, and even chromosomal rearrangement (26–28). MDR1/P-gp expression is also regulated by epigenetic mechanisms, such as methylation and acetylation (29). Post-transcriptional regulation of MDR1/P-gp expression by microRNA has also been reported (30,31). Recently, a potential transcriptional regulatory role of Twist1 has been identified in chemotherapy drug resistance. During generation of acquired resistance to paclitaxel, nasopharyngeal carcinoma cells showed upregulation of Twist1 at both the mRNA and protein levels and were also cross-resistant to vincristine (18). In the present study, our findings indicate a novel role of Twist1 in maintaining the cisplatin-resistant phenotype of cervical cancer HeLa cells through regulating MDR1/P-gp expression. However, further research is needed to define the molecular regulatory mechanisms.

Hypoxia is well known to induce resistance to drugs and radiation in solid tumors and a number of studies have indicated a direct regulation of Twist1 by HIF-1α (32). Ding *et al* found that there was a positive correlation between HIF-1α and MDR1/P-gp expression in colon carcinoma (33). Additionally, HNE1-T3 cells that acquired paclitaxel-resistant were reported to show a high amplification of the *Twist1* gene, decreased p53 and p21^Waf1^, but only a moderate alteration in MDR1/P-gp, which indicated that the Twist1-induced drug resistance results from interference with p53-related pathways (18). Furthermore, Cheng *et al* demonstrated that AKT2 is a downstream target of Twist1 and AKT2 is responsible for at least in part the Twist1-mediated paclitaxel resistance of breast cancer MCF-7 cells (20). Thus, the involvement of HIF-1α, p53 and AKT2 in the Twist1/MDR1-mediated cisplatin-resistant phenotype of cervical cancer will be investigated in our further studies.

In conclusion, this study provides the first evidence that Twist1 expression was significantly positively associated with MDR1/P-gp expression in human cervical cancer and that Twist1-mediated modulation of MDR1/P-gp expression plays an important role in the sensitization of cervical cancer cells to cisplatin treatment. Our results indicate a novel therapeutic strategy to overcome drug-resistance through inactivation of Twist1 expression in cervical cancer.

## Figures and Tables

**Table I:** Correlation analysis between MDR1/P-gp and Twist1 expression in human cervical carcinoma tissues.

	MDR1/P-gp		
		
Twist1	Positive	Negative	Total
Positive	24	7	31
Negative	3	8	11
Total	27	15	42

R=0.460, P=0.008.
